# Determination of metabolic activity in planktonic and biofilm cells of *Mycoplasma fermentans* and *Mycoplasma pneumoniae* by nuclear magnetic resonance

**DOI:** 10.1038/s41598-021-84326-2

**Published:** 2021-03-11

**Authors:** Ammar A. Awadh, Adam Le Gresley, Gary Forster-Wilkins, Alison F. Kelly, Mark D. Fielder

**Affiliations:** School of Life Sciences, Pharmacy and Chemistry, SEC Faculty, Kingston University London, Kingston Upon Thames, UK

**Keywords:** Biochemistry, Microbiology, Physiology

## Abstract

Mycoplasmas are fastidious microorganisms, typically characterised by their restricted metabolism and minimalist genome. Although there is reported evidence that some mycoplasmas can develop biofilms little is known about any differences in metabolism in these organisms between the growth states. A systematic metabolomics approach may help clarify differences associated between planktonic and biofilm associated mycoplasmas. In the current study, the metabolomics of two different mycoplasmas of clinical importance (*Mycoplasma pneumoniae* and *Mycoplasma fermentans*) were examined using a novel approach involving nuclear magnetic resonance spectroscopy and principle component analysis. Characterisation of metabolic changes was facilitated through the generation of high-density metabolite data and diffusion-ordered spectroscopy that provided the size and structural information of the molecules under examination. This enabled the discrimination between biofilms and planktonic states for the metabolomic profiles of both organisms. This work identified clear biofilm/planktonic differences in metabolite composition for both clinical mycoplasmas and the outcomes serve to establish a baseline understanding of the changes in metabolism observed in these pathogens in their different growth states. This may offer insight into how these organisms are capable of exploiting and persisting in different niches and so facilitate their survival in the clinical setting.

## Introduction

Mycoplasmas are facultatively anaerobic mollicutes that require an enriched growth media supplemented with nucleic acid precursors, fatty acids as well as amino acids to sustain their culture in laboratory conditions. These organisms are therefore considered to have a minimal genome^1^. Mycoplasmas are typically divided into fermentative and non-fermentative groups according to their ability to produce acid from glucose and degrade arginine respectively^2,3^. When grown under anaerobic conditions, lactate is the end product of the glucose oxidation, whereas under aerobic conditions, partial oxidation occurs and acetate and carbon dioxide (CO_2_) are formed^4^. Glucose and arginine are the substrates that are utilised as energy sources for mycoplasmas; thus, the majority of strains may be characterised according to whether they produce acid from glucose metabolism, ammonia from arginine hydrolysis or carry out both or neither of these reactions^5^. Amino acids, fatty acids, metabolic precursors and even cholesterol are essential growth factors and play a vital role in mycoplasma survival^6^. Due to the inability of mycoplasmas to synthesise macromolecules, they are dependent upon the nutrients found in host cells during infection^5,7^. Mycoplasma survival can be enhanced by biofilm formation^8,9^ and as a result, biofilms tend to be resistant to environmental conditions and host immune responses, relative to their planktonic counterparts^8–10^.

The view proposed by^11,12^ suggested that bacteria (potentially including mycoplasmas) attached to surfaces have differing metabolic activities compared to their planktonic counterparts due to physiochemical conditions and physiological characteristics expressed as a result of their growth. This phenomenon has been shown in biofilms produced by *Vibrio parahaemolyticus*, where a variety of cellular process including energy metabolism and biofilm development are influenced by certain polysaccharides^13^. In order to better understand mycoplasma biofilms and their influence in disease a comprehensive characterisation of their differing metabolic states is necessary within this complex cellular community. Metabolomics can provide such a systematic approach helping to clarify the complex mechanistics of biofilm states when compared with their free-living planktonic counterparts^14,15^.

In the current study, NMR spectroscopy in conjunction with principle component analysis (PCA) was used to examine the mycoplasma metabolome (both planktonic and biofilm) and characterise metabolic changes in mycoplasma species. However, as the cellular metabolome normally incorporates thousands of metabolites it was not possible to exhaustively analyse every individual component. In order to manage the analysis of these metabolites, chemomx software was utilised to identify the major features within NMR spectrum, such as the absence or presence of peaks, the change in the shape of peak intensity, or the change in chemical shifts that distinguish between the two classes^16,17^.

One-dimensional proton NMR (1D ^1^H NMR) is commonly used for global analysis of a metabolome^18–23^. These experiments were able to identify the network of resonances associated with specific metabolites through J-coupling in a model system^24,25^. The current study also used diffusion ordered spectroscopy (DOSY) NMR methodology to study the metabolome of planktonic and biofilm states for human mycoplasma preparations, providing size and structural information about the molecules therein^26^. In DOSY- NMR, the diffusion motions of the components in samples are encoded and decoded using the application of a set of pulsed field gradients (PFG)^27–31^. A particularly novel application of DOSY NMR is the capacity for spectral binning and principal component analysis (PCA) of the diffusion coefficients for different regions. This is the first time this technique has been employed in mycoplasmal metabolome characterisation and significant metabolic changes between biofilm and planktonic mycoplasmas have been determined.

## Materials and methods

### Mycoplasma strains used in the study

A selection of clinical strains from the Kingston University culture collection were utilised in this study including eight clinical strains of *Mycoplasma pneumoniae* all isolated from human sputum samples and eight *Mycoplasma fermentans* strains isolated from a Kaposi’s sarcoma patient, joint fluid (2 isolates), respiratory tract (2 isolates), urine, urethra and a cell line isolate. All strains were stored as freeze-dried cultures and were grown and sub-cultured in fresh Eaton’s broth medium.

### Growth of planktonic cells samples in liquid broth medium for *M. pneumoniae* and *M. fermentans* strains

Isolates of both *M. fermentans* and *M. pneumoniae* were inoculated into Eaton’s broth medium (at a cell density of 10^6^ cfu/ml) and incubated at 37 °C for 3–7 days to grow planktonic cells, whilst *M. pneumoniae* strains were incubated at 37 °C for approximately 30 days.

### Growth of biofilm samples in liquid broth medium for *M. pneumoniae* and *M. fermentans* strains

Aliquots (2 ml) of *M. fermentans* and *M. pneumoniae* (at a cell density of 10^6^ cfu/ml) were inoculated into 10 ml tissue culture flasks (Nunc, Fisher Scientific, UK) containing Eaton’s broth medium, and then incubated as previously described. Following incubation, biofilms were acquired from the flask using a cell scraper (Becton, Dickenson Company, BD, UK) and aliquoted into sterile bijous for processing.

### General NMR experimental for analysis of mycoplasma biofilm and planktonic serum extracts

A Bruker Avance III 600 MHz NMR spectrometer with 5 mm TXI Probe and temperature control unit was used for all 1D ^1^H NMR experiments. 5 mm Bruker Single Use NMR tubes (serum and DOSY). All spectra were acquired on Topspin 3.1 (Bruker, Germany). All NMR experiments were carried out at 25 °C. The Pulsecal routine was run prior to all experiments and the pulse angles adjusted accordingly for total correlated spectroscopy (TOCSY) and diffusion ordered spectroscopy (DOSY) experiments.

^1^H spectra for serum samples were acquired using 65,536 complex data points over a sweep width of 20.57 ppm using a pre-saturation of the water signal at 4.7 ppm and one spoil gradient, Relaxation delay (d1) was set to 10 s with the RGA set to 256 for quantitation (See Supplementary Information S1 for example). Serum samples had pH of 7.4 and a 700 μl aliquot was diluted with 300 μl D_2_O.

Diffusion spectra were obtained over 64 K data points (SW 10.3112 ppm) using longitudinal eddy current delay bipolar pulsed field gradient with 2 spoil gradients and pre-saturation sequence (LEDBPGPPR2s). This was used to obtain the diffusion series with δ = 4.6 ms and $\Delta$ = 125 ms. The relaxation delay was set to 4 s and the diffusion ramp consisted of 64 linear gradient steps, from 2 to 95% gradient intensity, each consisting of 16 scans.

Diffusion data was processed using a sine bell shaped window function phase over all data points prior to Fourier transformation (16,384 points) using Topspin 3.0 (Bruker, Germany). Diffusion data was processed using DOSY Toolbox, created by Mathias Nilsson, Manchester University. Individual peaks were fitted exponentially after a 2nd order polynomial baseline correction was employed^31^.

Errors in diffusion coefficient were calculated based on the Standard Deviation for each diffusion curve and are in line with the estimated error as reported for a similar mixture of *ca* 0.1 × 10^–10^ m^2^ s^−1^ ^31^. The Residual Sum of Squares for each of the diffusion curves is less than 5 × 10^–3^ in all cases.

### 1D ^1^H NMR evaluation of *Mycoplasma fermentans* and *Mycoplasma pneumoniae* in the serum based spent culture media

All 1D datasets were processed using Chenomx (Chenomx NMR Suite, Chenomx, Alberta, Canada). As has recently been reported^29^, internal reference standards such as trimethylsilyl propionate (TSP) can often complex with proteins in the analysis and it is highly questionable that reliable direct quantitation of individual metabolites is possible in such a mixture. The relative amounts of individual metabolites were of greater interest in a qualitative sense.

Metabolites used in the PCA were restricted to those for which a concentration was detected by Chenomx in all samples. All data points were normalised (area) using Unscrambler 10, using a constant weighting and cross validation.

### DOSY NMR evaluation of *Mycoplasma fermentans* and *Mycoplasma pneumoniae* in the serum based spent culture media

NMR spectra of metabolite mixtures are by their very nature complex and it was hypothesised that further information about metabolite mobility could be obtained using Diffusion Ordered Spectroscopy (DOSY). In addition, 1D NMR data provides many overlapping signals and while the Chenomx software is good at resolving some of the well-known metabolites, there was also a desire to investigate the existence of certain unknown metabolites. Through the use of a range of known metabolites as internal mass/diffusion standards it is possible to plot a LogMr vs LogD graph to calculate the approximate mass of an unknown and this has been used to good effect in complex mixtures of this type^30^.

Trying to automatically calculate diffusion coefficients from a pseudo 2D plot is not easily achieved and neither Amix nor Dosytoolbox are capable of dealing with the raw data to provide this output. DOSY spectra for both mycoplasmas were obtained for biofilm and planktonic samples in serum.

Therefore, the following process was adopted;

1. All DOSY spectra were calibrated with respect to both chemical shift and also diffusion, using internal standards of acetate, glucose, alanine and tyrosine. The range of molecular mass (Mr) afforded by this range of metabolites would enable the identification of an unknown Mr with good linearity (R^2^ > 0.95)
2. The individual spectra were binned (515 bins) with 0.02 ppm increments and all signal intensities for each bin for each of the 64 gradient incremented spectra.
3. The diffusion coefficients for each bin were calculated using an Excel curve-fitting algorithm.
4. The above three steps were carried out for each spectrum.
5. Using the 8 samples for each of the strains of mycoplasma we obtained a spreadsheet 8 column wide by 515 rows long with the matrix showing the diffusion coefficients for each bin for each sample. As water suppression was used the area of suppression was discounted from the spreadsheet.
6. For each strain of mycoplasma, the spreadsheet from 5 was analysed using Multibase 2015 addon to Excel and any differences analysed using PCA.

### Metabolite identification

Using Chenomx Profiler (Chenomx NMR Suite, Chenomx, Alberta, Canada) 1D processed datasets were analysed and the components assigned with the aid of TOCSY where appropriate. The pH was verified for each of the samples prior to processing so no internal pH standards were used and the line width and shifts were calibrated to formate as per the standard procedure for Chenomx data processing. Where assignment was ambiguous DOSY NMR was used to approximate the relative molecular mass (Mr) for individual components as previously shown^30^.

### Ethics approval

This research raised no ethical concerns for consideration as no human or animal subjects included in the experimental work.

## Results

### 1D ^1^H NMR evaluation of *Mycoplasma fermentans grown* in serum containing medium

**Figure 1:**
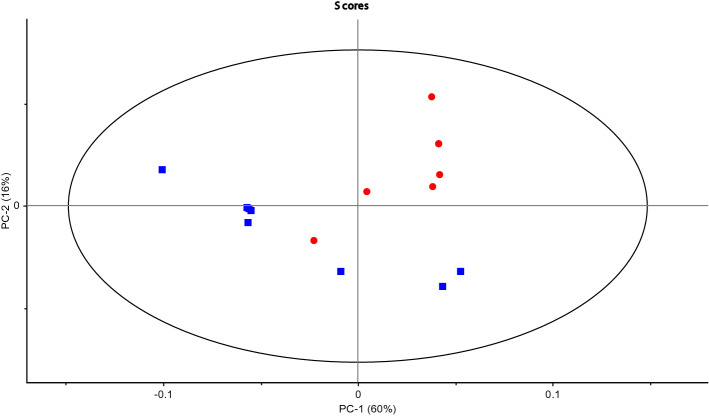
PCA scores plot for *Mycoplasma fermentans* (8 biofilm red filled circle and 8 planktonic blue filled square samples) from 1D ^1^H NMR analysis of serum.

**Figure 2:**
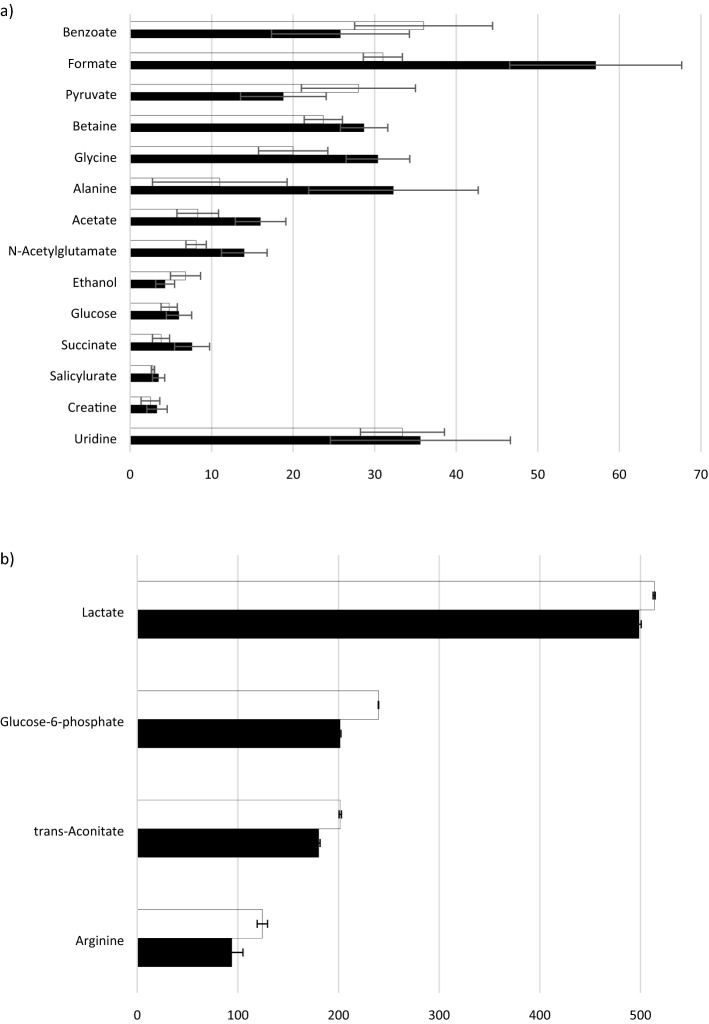
Black square planktonic and black filled square biofilm (**a**) Low concentration range 0–100 mM (**b**) High concentration range 0–500 mM. Summary of the identified metabolites in *Mycoplasma fermentans* serum contributing an observed variation in component signal intensity of > 10% *p* = 0.05. Metabolites were assigned using Chemnomx Profiler. Certain duplicate signals (owing to multiplicity of single components in 1D ^1^H NMR) have been removed and the signal intensities averaged).

From a statistical perspective, both biofilm and planktonic samples of *M fermentans* were considered in isolation. Following the same processing parameters described previously in the methodology section, PCA analysis was carried out (Fig. 1). These data indicate statistically significant discrimination between biofilm and planktonic *Mycoplasma fermentans* based on 1D ^1^H NMR analysis and multivariate treatment for the first time in complex serum containing growth media such as Eatons medium (Fig. 2).

**Figure 3:**
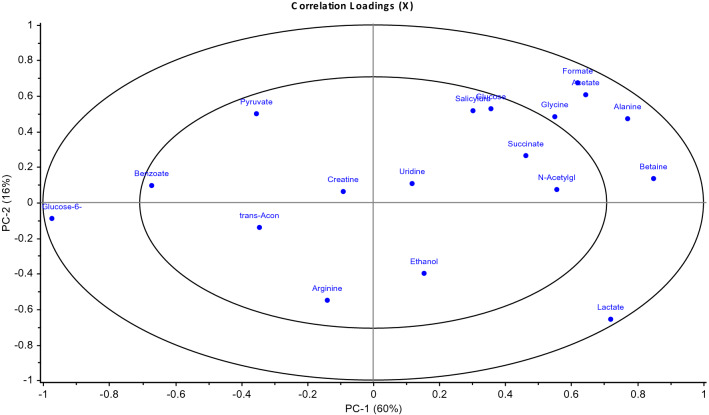
PCA Loadings plot for *Mycoplasma fermentans* from 1D ^1^H NMR analysis of serum. REMSEC value for y variable = 0.244.

According to the loadings plot (Fig. 3) it was possible to select those metabolites, which overall explained more than 50% of the variation observed in the 1D ^1^H NMR data and where there was a > 10% difference in the mean intensity of the signal for a metabolite between biofilm and planktonic NMR spectra (Fig. 2).

The loadings plot explained more than 50% of the variation observed in the 1D ^1^H NMR data and where there was a > 10% difference in the mean intensity of the signal for a metabolite between biofilm and planktonic NMR spectra.

The concentration values were obtained for a cluster plot for accuracy and the signals were correlated to the individual metabolites that are described in the Chemnomx process in metabolite identification section in methodology.

As with all data analysed by unsupervised multivariant methods, the robustness of the data is directly correlated to the number of samples analysed (Supplementary Information S2).

The current study has shown discriminative patterns of metabolites for planktonic and biofilm cells of eight given strains of *M. pneumoniae* and *M. fermentans*. However, further samples and replicates would be necessary to re-enforce the power of this technique; moreover, further work would be useful to substantiate the significance of these results as they stand.

### 1D ^1^H NMR evaluation of *Mycoplasma pneumoniae* in the serum based spent culture media of both biofilm and planktonic growth conditions

**Figure 4:**
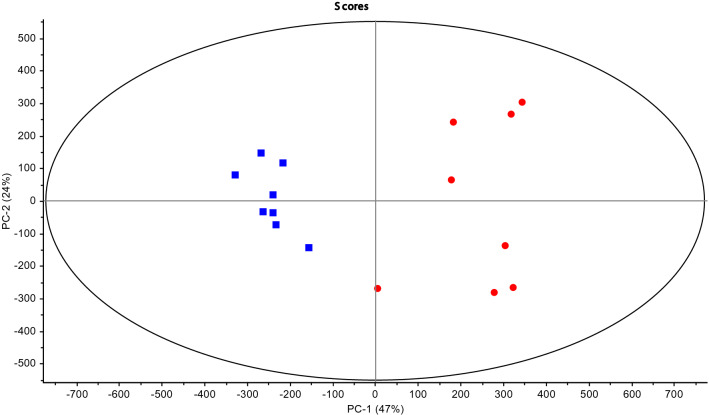
PCA Scores Plot for *Mycoplasma pneumoniae* (8 biofilm blue filled square and 8 planktonic red filled circle samples) from 1D ^1^H NMR analysis of serum.

Both biofilm and planktonic samples of *M. pneumoniae* were considered in isolation. Following the same processing parameters that were described previously in the methodology section, PCA analysis was carried out indicating a statistically significant difference between the serum composition of biofilm and planktonic arrangements of *M. pneumoniae* (Fig. 4)*.*

**Figure 5:**
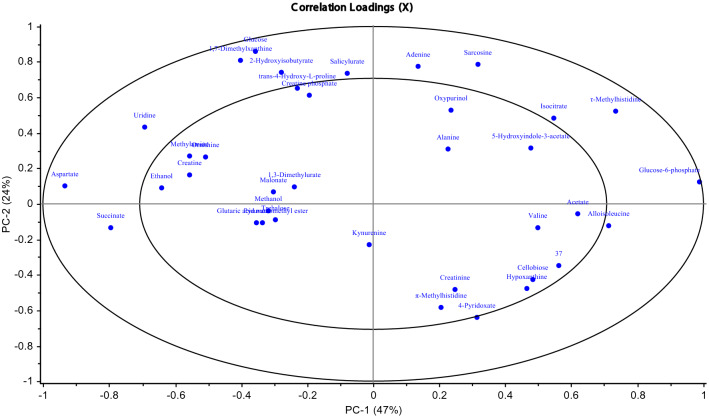
PCA Loadings plot for *Mycoplasma pneumoniae* from 1D ^1^H NMR analysis of serum. REMSEC value for y variable = 0.252.

**Figure 6:**
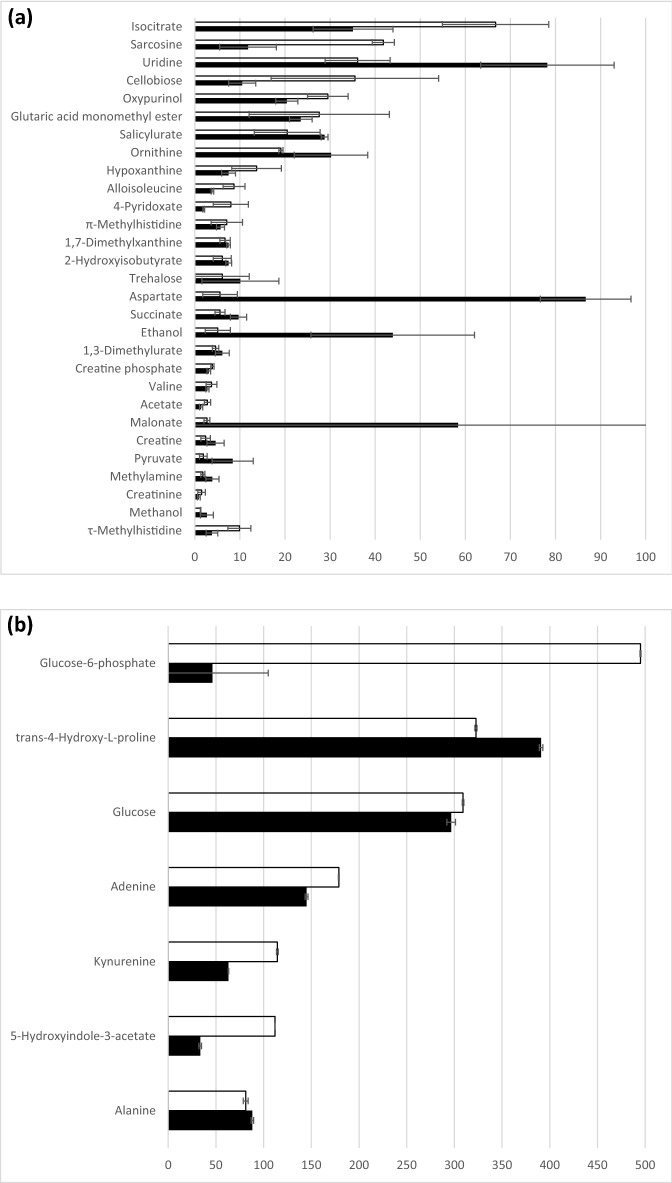
Black square planktonic and black filled square biofilm. (**a**) Low concentration range 0–100 mM (**b**) High concentration range 0–500 mM. Summary of the identified metabolites in *Mycoplasma pneumoniae* serum contributing an observed variation in component signal intensity of > 10% *p* = 0.05. Metabolites were assigned using Chemnomx Profiler. Certain duplicate signals (owing to multiplicity of single components in 1D ^1^H NMR) have been removed and the signal intensities averaged).

Using the loadings plot (Fig. 5), it was possible to select specific metabolites, which explained more than 50% of the variation observed in the 1D ^1^H NMR data and where there was a > 10% difference in the mean intensity of the signal for a metabolite between biofilm and planktonic NMR spectra (Supporting Information S3). The media components used in this study (Eaton’s media) in the absence of any *Mycoplasma* species, were also identified by 1D ^1^H NMR in order to provide baseline recognition of known metabolites, Chenomx software was used to analyse and identify the different metabolite components (Fig. 6).

### Diffusion NMR analysis of *Mycoplasma pneumoniae*

**Figure 7:**
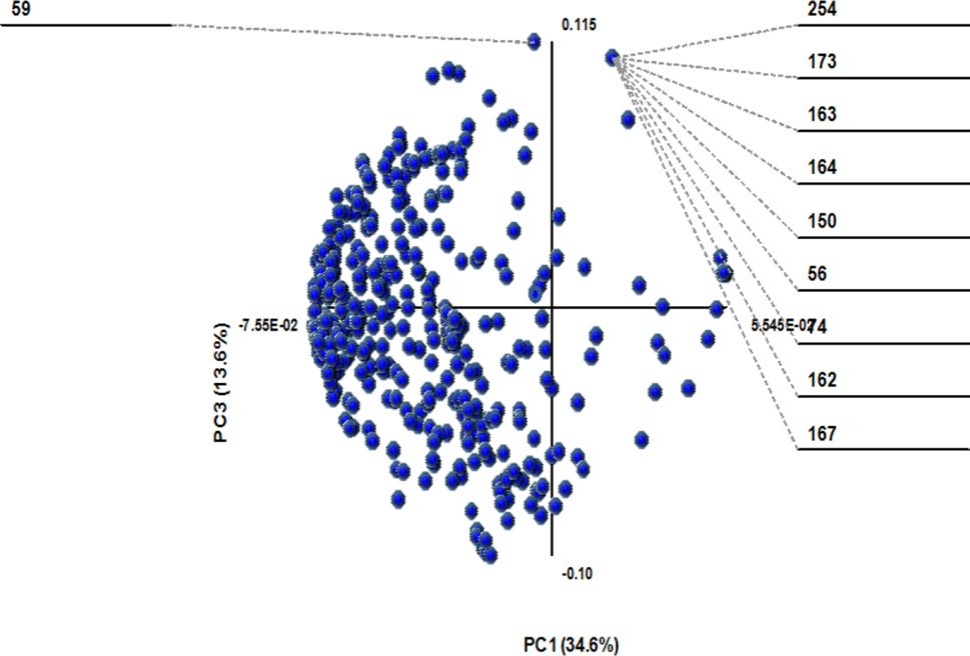
PCA scores plot analysis of the diffusion coefficients for 490 bins for *Mycoplasma pneumoniae.* 95% of the variation is explained by 3 PCs. With R^2^ = 0.60 and Q^2^ = 0.75 as the cumulative result of the 3 PCs.

The data was processed using MS Excel with the “Multibase 2015” add on and no weighting was applied to the variables since there is no precedent in the literature to aid explanation of the data and future investigations may be required to consider all possible implications of differing diffusion coefficients. The PCA analysis for *M. pneumoniae* shows that some of the variation between datasets could be explained using diffusion coefficient data. The fact that 3 PCs are required to explain 95% of variation implies a certain degree of complexity in the way the data can show differences between biofilm and planktonic mycoplasmas and further investigation as to how this correlates to metabolite amplification is beyond the scope of the current study and requires further work (Fig. 7).

**Figure 8:**
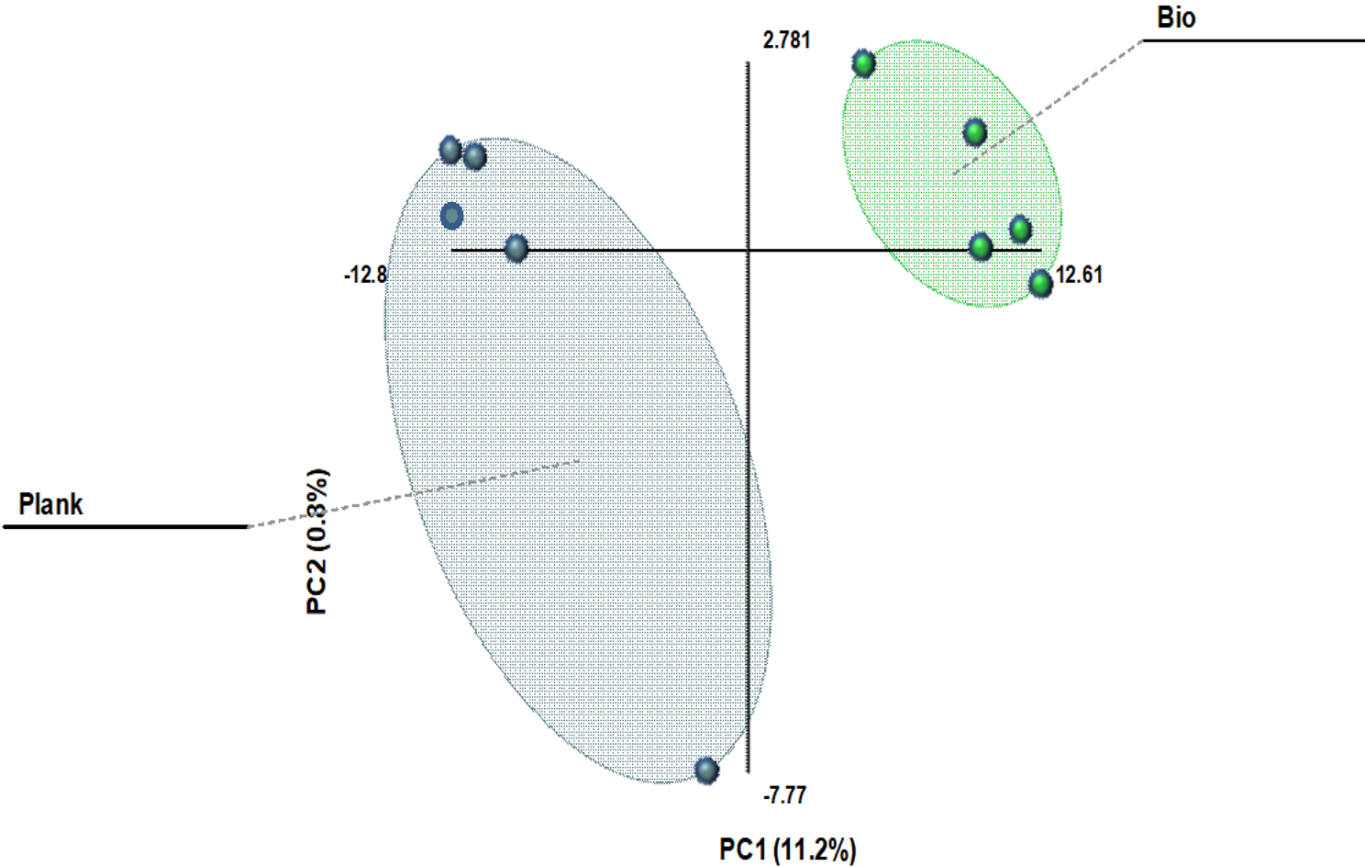
PCA scores plot for diffusion data for *Mycoplasma pneumoniae.* PC1 describes the majority of the variation observed.

The PCA was carried out in order to identify which components had the most discriminatory aptitude (Fig. 8). Whilst the majority of signals were irrelevant with regards to the data set, it was possible to identify regions of the spectra which were correlated with the biofilm samples and those which correlated to the planktonic (Supporting information S4).

The large number of metabolites appear to diffuse in a substantially different fashion making it a challenge to identify any one core component which accounts for the observed variation. However, some general observations can be made for example, that components at around 1 ppm seem to diffuse faster for biofilm mycoplasmas when compared to planktonic mycoplasmas (Supporting Inofrmation S5). Further work would be required to automate the correlation of the signals with a specific component. The introduction of operator assignment error when the bin size is as small as it is could easily lead to incorrect assignment of a signal to a specific metabolite.

### Diffusion NMR analysis of *Mycoplasma fermentans*

PCA analysis of the diffusion data for *M. fermentans* initially showed little grouping in the scores plot and this was confirmed by the statistical output, which showed a negative value for Q^2^ and poor statistical explanation of the data. The residual variation was too high for this model and therefore further interrogation would have yielded unreliable pattern data (Supplementary Information S6), meaning this approach is not worth pursuing without optimisation on simpler systems.

## Discussion

Mycoplasmas are characterised by limited biosynthetic capabilities, largely due to their minimal genome^32,33^. Loss of genes involved in biosynthesis of lipids, amino acids, vitamins and cofactors during their evolution^34,35^ has resulted in a reliance on external nutrients from host cells in vivo or rich media in vitro^8,34^. Furthermore, *Mycoplasma* species are often deficient in intermediary energy metabolism, resulting in a lifestyle strictly dependent on the natural host^36^. Reliance upon host cells for biosynthetic precursors may lead to competition and therefore disrupt host cell function and integrity^35^.

Mycoplasmas are divided into fermentative and non-fermentative organisms^2^ according to their ability to catabolise carbohydrates via the phosphoenolpyruvate phosphotransferase (PEP-PTS) system^37^. Glucose, and other fermentable sugars, are a crucial energy source, especially for the fermentative mollicutes. Glucose may also act as a precursor for the synthesis of other saccharides that stimulate the growth rate of mollicutes^38–40^.

Host cell attachment by mycoplasmas, as a biofilm or part thereof, and cytotoxic metabolite production may contribute towards significant host cell damage, commonly observed in infection with these pathogens. Biofilm formation also frequently results in increased resistance to antimicrobial agents and persistence of many organisms including *M. fermentans* and *M. pneumoniae*^41–43^. Physiological characterisation of biofilms is therefore vital to develop effective anti-mycoplasma treatments^25^.

Although global metabolite analysis has been proven as a powerful tool for understanding bacterial physiology^26,27^ the work presented here is the first DOSY-NMR metabolomics-based study in human mycoplasmas.

NMR-based metabolomics is a relatively new technology in biological applications and correspondingly has seen limited exploration in the investigation of microbial biofilms^17^. The purpose of the evaluation of biofilm and planktonic mycoplasmas using 1D ^1^H NMR was to establish the amplification of one or more metabolites/media components (markers) dependent on the mycoplasma being grown either as a biofilm or in planktonic state. The use of 1D NMR techniques for the analysis of serum and also cytosolic contents are well established^25,28,31^ and analysis of *Acinetobacter baumannii* strains using 1D NMR, 2D TOCSY and PCA analysis demonstrate the usefulness of these types of investigations in potentially elucidating details of the different metabolic pathways. The aim of the current research was to use these techniques to identify differences in metabolite composition for *M. pneumoniae* and *M. fermentans* in both biofilm and planktonic forming conditions. In addition, we aimed to evaluate the potential of Diffusion Ordered Spectroscopy (DOSY) to assist in discriminating between planktonic and biofilm mycoplasmas and what significance these points of discrimination may have, either to assist in the classification of a mycoplasma as either biofilm or planktonic derived, or to provide more information about the constituents of the biofluid and potentially the differences in metabolic pathways. Additionally, this approach could also help to understand the potential changes in mycoplasmal metabolism under different growth conditions. The current study examines the overall metabolism of the organisms in the stated growth condition (planktonic or biofilm) and not the rate of metabolism. The measurement of bacterial metabolism for communities in a biofilm cannot reliably be measured and currently the metabolomic differences between the growth states as a whole is reported rather than growth rate measurements^44–48^.

In reference to carbohydrate metabolism. many mycoplamas lack hexokinase, therefore glucose is transported into cells and 'captured' by the phosphoenolpyruvate: phospho-transferase (PEP:PTS) system for degradation to pyruvate in the Embden–Meyerhof–Parnas (EMP) glycolytic pathway^49–54^. Oxidative phosphorylation deficient mycoplasmas can metabolise pyruvate via two alternate pathways to generate ATP: oxidation by pyruvate dehydrogenase to acetyl-CoA, followed by the action of phosphate transacetylase and acetate kinase to yield acetate and ATP. Alternatively, pyruvate is reduced to lactate by lactate dehyrdogenase with concomitant oxidation of NADH to NAD to facilitate continued ATP generation via the EMP^50,53^. Energy metabolism in mycoplasmas is therefore principally dependent on fermentation via the EMP to lactate under anaerobic conditions or acetate and CO_2_ under aerobic conditions^3,34^.

In *M. fermentans*, acetate was found to be greater in the biofilm suggesting that there is a bias toward the pyruvate dehydrogenase route for pyruvate metabolism. For both mycoplasma strains, the data indicates biofilms potentially produce more energy than planktonic cells. Glucose-6-phosphate was more abundant in planktonic cultures of both *M. fermentans* and *M. pneumoniae*, compared to biofilm serum (Figs. 2 and 6). Elevated uptake and 'capture' of glucose and metabolism to the end-products lactate and acetate is required in order to generate the ATP necessary for growth and cellular functions^32^, and thus facilitate the formation of biofilm.

The current study has identified ethanol (Figs. 2 and 6), an end-product of fermentative metabolism, as a metabolite; however, acetaldehyde which is the metabolic intermediate in ethanol formation from pyruvate was not identified. Similarly, alcohol dehydrogenase, the enzyme required for metabolism of pyruvate to ethanol was not experimentally detected in *Mycoplasma pneumoniae*. This observation concurs with *M. pneumoniae* proteomics data^32^; however, the current study measured ethanol secretion by mass spectrometry. The ethanol level was higher in biofilms than planktonic cells in *M. pneumoniae*, whereas in *M. fermentans* it was observed to be higher in planktonic cells. This suggests that the ability to synthesise ethanol has a greater importance in the growth of *M. pneumoniae* biofilms. From the analysis of large metabolic networks^55,56^, evidence from genetic studies and experimental investigations of protein abundance^57^ it is apparent that *Mycoplasma pneumoniae* lacks almost all anabolic pathways, including those for amino acid synthesis and metabolism^32^. Consequently, *M. pneumoniae* is dependent on the import of large amounts of amino acids^32^ and the requirement for enriched culture media supplemented with these^1^. In the current study, levels of amino acid metabolites were identified in both biofilms and their planktonic counterparts. Global analysis of *M. pneumoniae* metabolism indicated proteins mapped to the metabolic pathways in KEGG data base, including proline metabolic pathway (http://www.genome.jp/kegg/pathway/map/map00330.html). The results showed that the proline level (in the form of 4-Hydroxy-L-Proline) in *M. pneumoniae* was greater in biofilm cell serum compared to its planktonic counterpart (Figs. 2 and 6). This finding would suggest that proline iminopeptidase (Pip) may release proline from peptide and the aspartate-ammonia ligase (AsnA) catalyses the interconversion of aspartate and asparagine^33^, and thus may be crucial for mycoplasma survival.

In *M. fermentans*, “arginine and proline” amino acid metabolic pathway has been recognised using genomic studies^33^. *M. fermentans* produces ATP from arginine metabolism via the arginine dihydrolase (ADH) pathway, similar to that found in *M. hominis*^33,58^. The ADH pathway catalyses the conversion of arginine to ornithine, ATP, CO_2_ and ammonia. The current work showed that arginine levels in *Mycoplasma fermentans* were greater in planktonic cells than biofilms.

Alanine was more prominent in biofilm metabolism of *M. fermentans* compared to its planktonic growth state (Figs. 2 and 6). This amino acid could be crucial in the absence of pyridoxal pyrophospahte as it could be a preferred element in the supplemented media instead of tyrosine and phenylalanine for optimum growth, suggesting the presence of an operative shikimic acid pathway for aromatic amino acids synthesis^40^. Additionally, alanine was observed to be marginally greater in the biofilm of *M. pneumoniae* (Figs. 2 and 6), which may suggest that alanine was enriched in the cytosol due to the subsequent of cellular import from the surrounded growth medium^32^.

Additionally, metabolite profiles in the current study demonstrated the presence of glycine and betaine, which are found to be higher in biofilms when compared with planktonic cells of sera for *M. fermentans* (Figs. 2 and 6). Glycine is considered as a very efficient osmolyte found in a wide range of bacteria, where it is accumulated at high cytoplasmic concentration^58,59^ in order to build up an internal osmotic strength and prevent the diffusion of water out of the cells and thus maintain the cellular water content^59,60^. When bacteria are grown at an inhibitory osmolarity, the enzyme activities that lead to glycine and betaine degradation decrease, whereas the enzyme activities that convert choline to glycine betaine either remain constant or increase^61^. In this way, a high concentration of glycine betaine can be maintained in osmotically stressed cells^62^. Mycoplasmas in nature are normally exposed to different types of stresses including osmolarity shifts and their resistance to different stresses is noticed^63,64^. Consequently, the high level of these osmolytes in biofilm cells may indicate to their importance in growth and biofilm formation.

The metabolite profiles in the current study showed some interesting observations, such as the presence of valine and alloisoleucine in *M. pneumoniae* (Supplementary information S4 for *M. pneumoniae*)*.* Depending on the examination of the phylogenetic distribution of the enzymes participating in isoleucine/valine metabolic pathway, it was found that all the genes coding for the enzymes of isoleucine/valine biosynthesis were missing in *M. pneumoniae*^65^. Thus, the presence of these metabolites in the profile might be related to the high nutritional value and complexity of the surrounding growth medium. This study also identified the presence of creatine in the biofilm of both *M. fermentans* and *M. pneumoniae w*ere higher than their planktonic counterparts (Figs. 2 and 6). The presence of creatine is correlated to the biosynthetic pathway of arginine that involves the conversion of citrulline to arginine catalysed by arginosucciante synthase, which in turn provides the essential arginine for creatine biosynthesis. Creatine, the end product of the pathway, acts to conserve the utilisation of semi-essential amino acids, including arginine^66^. Although creatine biosynthesis in mycoplasmas is still elusive and not fully described, these findings could be connected to arginine metabolism, as described above.

In relation to nucleotide Metabolism, levels of Uridine were higher in the biofilm than planktonic state after for both *M. fermentans* and *M. pneumoniae* (Figs. 2 and 6) Uridine nucleic acid derivatives could be used as a precursor of polysaccharides, such as exopolysaccharide (EPS), which is important in the maintenance of biofilm structure^25^. In *Mycoplasma pneumoniae*, Uridine is involved in the Leloir pathway that comprises a step in galactose phosphorylation^67^. Furthermore, EPS production requires both free galactose and the Leloir pathway in order to form a biofilm^67^.

## Conclusion

This work is the first study to quantify a set of key metabolites, including glycolysis compounds, amino acids, and nucleotides, in the growth of human mycoplasmas. The results obtained suggested that metabolic pathways in human mycoplasmas, such as *M. fermentans* and *M. pneumoniae* were regulated by multiple enzymatic reactions in order to fulfil key metabolic activities. Therefore, protein abundance in the metabolic network could provide a qualitative picture of *M. fermentans* and *M. pneumoniae* metabolic pathway activity in both biofilm and planktonic cells under laboratory growth conditions. There are examples of how metabolomic differences have been observed in in planktonic and biofilm cells of *Mycoplasma genitalium*^46^, *Staphylococcus aureus*^44,45^, *Gardnerella vaginalis*^47^ and *Rhizobium alamii*^48^. Whilst these studies all discuss the difference in metabolomic profiles in the species discussed between planktonic and biofilm growth states, none look at the rate of metabolism. As there is a range of growth states throughout a biofilm from the apical to the basolateral surface measuring the rate of growth is widely viewed as not possible. As a result, studies often analyse the production of materials, compounds metabolic markers from the biofilm as a whole and not the rate of metabolism as this is not currently possible. Hence the data presented here is the first use of DOSY-NMR to examine the metabolism of these mycoplasmas in their planktonic and biofilm growth states, as a whole and not on a rate wise basis. The clarification of metabolic pathway activity in both biofilm and planktonic cells enables us to gain a better understanding of vital and non-essential metabolites involved in the formation and establishment of human mycoplasma biofilms.

## Supplementary Information

Supplementary Information 1.
