# Supplementary material for: Determination of metabolic activity in planktonic and biofilm cells of Mycoplasma fermentans and Mycoplasma pneumoniae by nuclear magnetic resonance
Source: Sci Rep. 2021 Mar 11;11:5650. doi: 10.1038/s41598-021-84326-2 (PMC7952918; doi:10.1038/s41598-021-84326-2)
Supplement: Supplementary file 1 — Supplementary Information 1. [file 41598_2021_84326_MOESM1_ESM.docx]

Determination of Metabolic Activity in Planktonic and Biofilm Cells of *Mycoplasma fermentans* and *Mycoplasma pneumoniae* by Nuclear Magnetic Resonance

Ammar A. Awadh, Adam Le Gresley, Gary Forster-Wilkins, Alison F. Kelly, Mark D. Fielder*.

School of Life Sciences, Pharmacy and Chemistry, SEC Faculty, Kingston University London, UK

**S1. ^1^H NMR of the extracellular metabolic profiles of serum from *Mycoplasma pneumoniae* biofilm cultures. In the NMR spectrum, valuable information could be obtained from a single peak including chemical shifts which is related to the local chemical environment of specific molecules (e.g. ^1^H). Importantly, the peak intensity is directly proportional to the metabolite’s concentration. Typically multiple distinct NMR resonances are observed for each molecule. Y-axis correlates to intensity. X-axis is chemical shift in ppm. Metabolites are assigned by their peak chemical shift and multiplicity.**


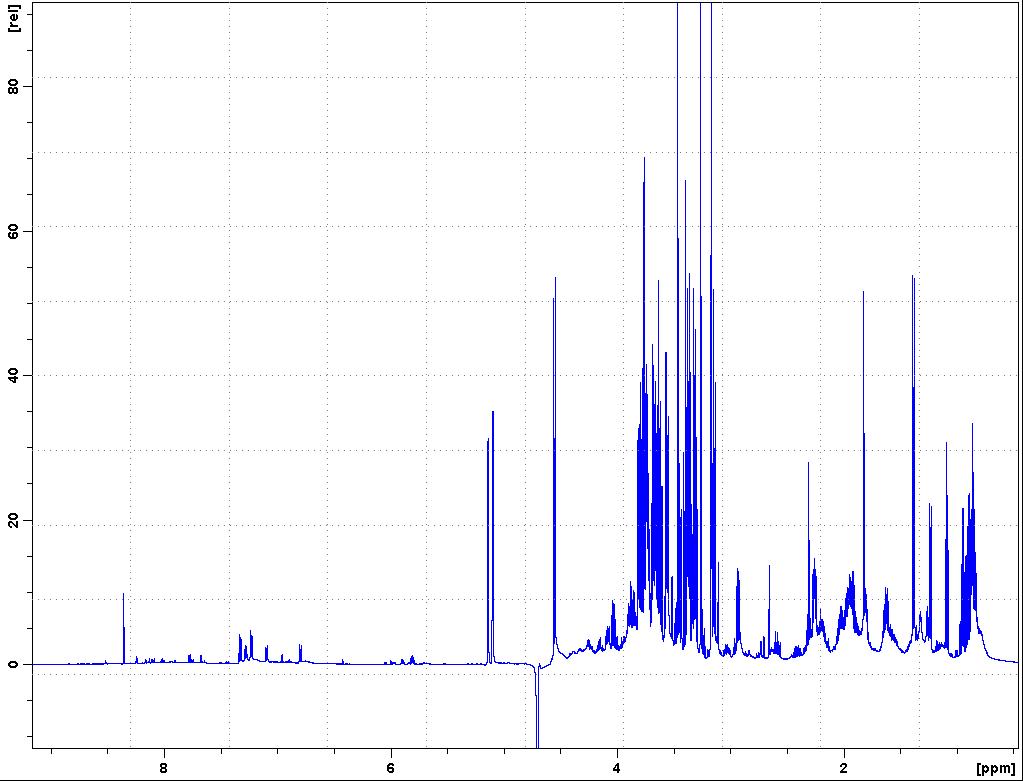


**S2 Explained vs Residual variance for *Mycoplasma fermentans* PCA (Normalised)**

**S3 Explained vs Residual variance for *Mycoplasma pneumoniae* PCA (Normalised)**

**S4 PCA loadings plot for Diffusion Data for *Mycoplasma pneumoniae***

**S5 Component contribution (Loading) to observed variation in diffusion coeffcient for each of the spectral bins. Positive correlation in the graph shows increased diffusion coeficcient of metabolites in biofilm whereas the negative values show increased diffusion coeffcient for metabolites in planktonic conditions. Y-axis units are arbitrary but represent the intensity of NMR signals for one type of *Mycoplasma* species when compraed to others and X-axis show reduced spectra bin count. Each bin is 0.02ppm in length. Axis is right to left 0-10ppm as a normal 1H NMR spectrum would appear.**


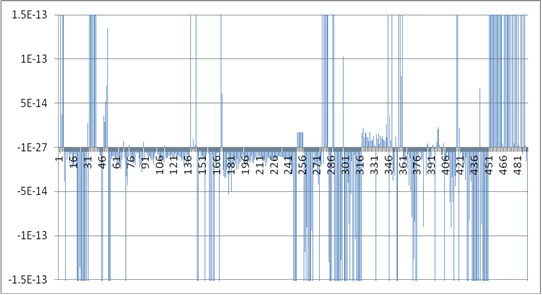


**S6 (Left) PCA analysis of diffusion coefficient variation between biofilm and planktonic *Mycoplasma fermentans* (Right) Significance output chart for variation. The poor description of the data by PCA may be down to the growth rate of the *Mycoplasma fermentans* and the pattern of data featuring not just growth conditions but also the stages of development.**

Q^2^

% values

R^2^
